# Convolutional Networks and Transformers for Mammography Classification: An Experimental Study

**DOI:** 10.3390/s23031229

**Published:** 2023-01-20

**Authors:** Marco Cantone, Claudio Marrocco, Francesco Tortorella, Alessandro Bria

**Affiliations:** 1 Department of Electrical and Information Engineering, University of Cassino and Southern Latium, 03043 Cassino, FR, Italy; 2 Department of Information and Electrical Engineering and Applied Mathematics, University of Salerno, 84084 Fisciano, SA, Italy

**Keywords:** mammography classification, convolutional networks, vision transformers, computer aided diagnosis

## Abstract

Convolutional Neural Networks (CNN) have received a large share of research in mammography image analysis due to their capability of extracting hierarchical features directly from raw data. Recently, Vision Transformers are emerging as viable alternative to CNNs in medical imaging, in some cases performing on par or better than their convolutional counterparts. In this work, we conduct an extensive experimental study to compare the most recent CNN and Vision Transformer architectures for whole mammograms classification. We selected, trained and tested 33 different models, 19 convolutional- and 14 transformer-based, on the largest publicly available mammography image database OMI-DB. We also performed an analysis of the performance at eight different image resolutions and considering all the individual lesion categories in isolation (masses, calcifications, focal asymmetries, architectural distortions). Our findings confirm the potential of visual transformers, which performed on par with traditional CNNs like ResNet, but at the same time show a superiority of modern convolutional networks like EfficientNet.

## 1. Introduction

Breast cancer is the most frequent malignancy in women worldwide. In 2020, approximately 2.3 million new cases were diagnosed and 685,000 women with breast cancer died [1]. The global incidence of this disease is increasing with a 3.1% annual rate, beginning with 641,000 cases in 1980 and reaching more than 1.6 million in 2010. This trend is likely to continue [2,3].

The process of the diagnosis of breast cancer starts with a mammography exam. Mammography is an imaging technique that uses low energy X-ray beams to examine the human breast for diagnosis and screening. The goal of mammography is the early detection of breast cancer, typically through the identification of specific lesions like masses, calcifications, architectural distortions or focal asymmetries. Early breast cancer is a type of cancer that has not spread to other parts of the body and is considered curable in around 70–80% of cases, whereas advanced metastatic cancer is not considered curable. For this reason, an early diagnosis is crucial to improve the life expectancy of patients. Many countries adopt population screening for breast cancer using mammography aimed at detecting the disease at an early stage to allow effective treatment. A frequent issue with mammography screening are false positive recalls which involve additional exams and would likely lead to overtreatment.

In order to assist radiologists interpretation of mammograms, Computer Aided Diagnosis (CAD) systems have been developed as an alternative to double reading, improving clinicians accuracy and patient outcome [4]. These systems were traditionally based on hand-crafted mathematical models designed by field experts using domain knowledge [5]. Through the 2010s, the field of visual recognition shifted from engineering features to designing Convolutional Neural Network (CNN) architectures [6,7]. This approach was later adopted in a number of medical image analysis applications [5,8,9], although there are still systems based on domain knowledge approaches [10]. It has been proven that CAD systems based on CNNs can be as accurate as experienced radiologists [11]. Furthermore, hybrid models averaging the probability of malignancy predicted by a radiologist with CAD predictions, can be more accurate than either of the two separately. The main advantages of CNNs are the capability of extracting hierarchical features from raw data and the embedded sliding window approach that is particularly suited for visual processing. CNNs have also several built-in inductive biases that make them work well on medical images, like translation equivariance and spatial locality. Compared with traditional systems, the black-box nature of CNNs results in a lack of explainability which is crucial for automated diagnosis. Furthermore CNNs are often computationally expensive to train, thus resulting in a high energy footprint.

CNNs have been adopted in many applications related to mammography [4,12], such as mass segmentation [13], mass detection [14,15,16], calcification detection [15,17], mammography classification [18,19], classification of pre-segmented masses [20]. Most of these works use digitized screen-film mammograms datasets like the Digital Database for Screening Mammography (DDSM) [21], consisting of 2620 images, or InBreast [22] which consists of only 410 full-field digital mammograms, or both. In 2019 a comparative study [23] reported the performance of eight deep convolutional neural networks in the context of breast mass classification into benign or malignant on DDSM-400 and CBIS-DDSM datasets [21,24]. This study shows that networks trained in a fine-tuning scenario, initialized with the pre-trained weights from the ImageNet dataset [25] and then fine-tuned on a mammography dataset, obtained a significant increase in performance compared to training from scratch. Specifically, the best result on DDSM-400 was obtained by a ResNet-101 [26] with an AUC of 85.9%, while the best result on CBIS-DDSM was obtained by a ResNet-50 with an AUC of 80.4%.

Recently, the Vision Transformer (ViT) [27] derived from the adaption of the vanilla Transformer [28] from Natural Language Processing (NLP) domain to computer vision. ViT achieved state-of-the-art classification performance in natural image domain at the cost of being trained with hundreds of millions of images. Unlike CNNs, ViT lacks some inductive biases and has the ability of encoding long-range dependencies in the early layers. To counteract the weakness of ViT, other transformer-based networks have been proposed. DeiT [29] seeks to mitigate the strong data demand through the use of a more efficient training strategy and aggressive data augmentation. Swin [30], SwinV2 [31] and NesT [32] adopt a hierarchical approach to reduce the computational complexity of classical transformers, this also reintroduces some priors typical to CNNs.

The medical imaging field has also witnessed growing interest for transformers and their characteristic to capture global context compared to CNNs with local receptive fields [33,34,35]. Currently, the literature using transformer-based networks for mammography analysis is still limited, especially in the case of whole image classification. Very recently, Chen et al. [36] proposed a transformer-based method to classify multi-view mammograms, that achieve an AUC of 0.818 on a dataset consisting of 3796 images, surpassing the state-of-the-art multi-view CNN model. Simultaneously with our research, Swin transformer has been tested in single-view mammography classification obtaining an AUC of 0.722 on DDSM dataset [37]. To the best of our knowledge, no research uses the NesT architecture in any task related to mammography.

In this experimental study we compare 33 distinct network models belonging to eight different families: ResNet [26], DenseNet [38], EfficientNet [39] and ConvNext [6] for the convolutional paradigm and ViT [27], DeiT [29], SwinV2 [30,31] and NesT [32] for transformer-based models. The analysis was performed on the task of binary classification of single-view whole mammograms where one class consists of normal images with no abnormalities and the other of images containing malignant findings.

**Figure 1:**
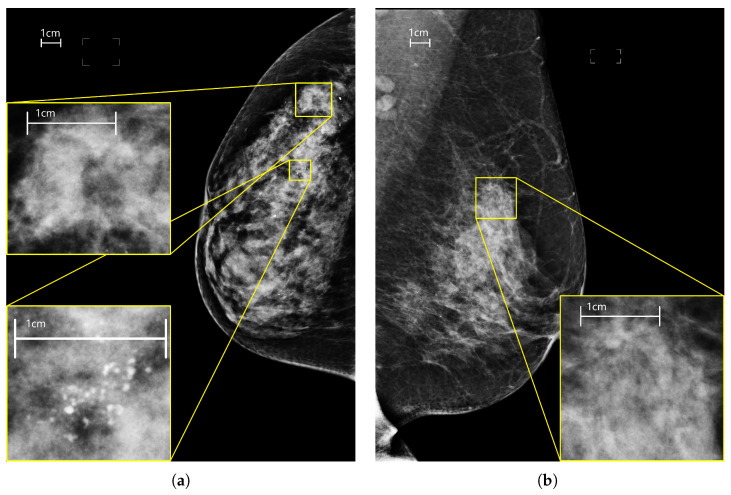

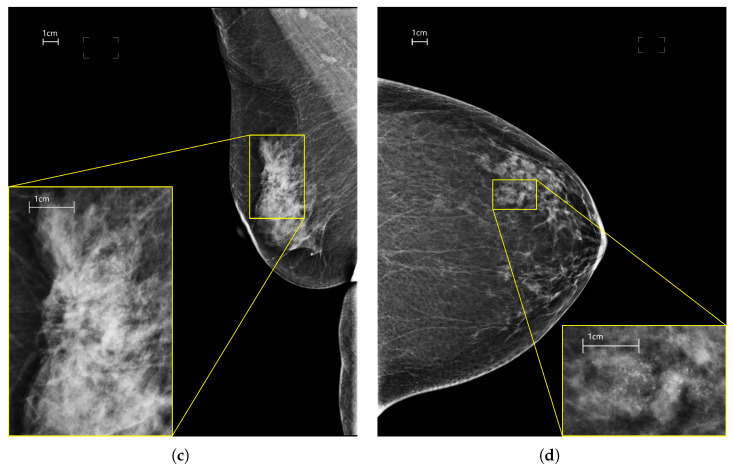
Mammograms of different views, laterality and containing different lesions. (**a**) right cranio-caudal with two lesions: a calcification cluster and a mass. (**b**) left medio-lateral oblique with a focal asimmetry. (**c**) right medio-lateral oblique with an architectural distorsion. (**d**) left cranio-caudal containing a mass with calcifications inside.

Lesions have different characteristics in shape, appearance, size and sparseness: masses are space-occupying lesions with a typical diameter of a few centimeters; calcifications are tiny deposits of calcium with a dimension typically less than a millimeter almost always grouped in a cluster; asymmetries are defined as unilateral deposits of fibroglandular tissue; architectural distortions refer to disruptions of the normal pattern of tissue with no definite mass visible. In Figure 1 mammograms with different types of lesions are shown. In this context, we want to analyze the performance of CNNs and transformers, which are based on two different paradigms (locality and global attention), with respect to lesions with different spatial patterns (dense and sparse). Other characteristics of the lesions, especially the size, make the input images resolution a crucial factor for the detectability of malignant findings and the correct classification of mammograms. Therefore we conducted a series of experiments by varying the input resolution from 256×128 to 2048×1024 with a 128-pixel step on the image width using two networks, an EfficientNet-B0 and a SwinV2-B. For each experiment, we also computed per-lesion metrics to highlight the correlation between input resolution, performance by type of lesion, and network architecture. Our major contributions are:

- we compare the performance of 14 transformers and 19 CNNs on the classification of mammograms containing lesions with different characteristics in terms of size, shape, texture, and sparsity;
- we analyze the performance of all networks with respect to the type of lesion present in the images;
- we perform an experimental comparison using eight different input resolutions;
- this is the first experimental study that uses transformers-based architectures and compares them with convolutional-based models for mammography classification applied on a large database of full-field digital mammography images.

The rest of this work is structured as follows. In Section 2 we provide the detailed description of the dataset and the experimental setup, we also briefly discuss the deep learning network architectures and metrics used. In Section 3 the obtained results by varying network architectures and image resolutions are presented. Section 4 discusses the results, we also present the limitations of this experimental study and a brief discussion on explainaibility. In Section 5, we conclude with a summary, a critical discussion and some guidelines for interested researchers.

## 2. Materials and Methods

### 2.1. Dataset

The OPTIMAM Medical Image Database (OMI-DB) is a large database of over 2.5 million full-field digital images from over 170,000 women enrolled in three UK breast screening centers in the years 2010–2020. For this work, we made an agreement with the OPTIMAM steering committee obtaining a subset of OMI-DB consisting of 148,460 images from 6000 women. This includes 1030 women with normal breasts, 970 women with benign findings, 3970 women with screening-detected cancers and 30 women with interval cancers, and a total of 8583 expert annotations on 7925 images. Each image may contain several annotations and each lesion may be of several types simultaneously. For example, a single image could contain a focal asymmetry and a mass with calcifications inside. The database comprises both *for processing* and *for presentation* mammograms from different vendors including: Hologic Inc., Siemens, Philips, General Electric Medical System, and Bioptics Inc. For this study, we selected only *for presentation* mammograms from Hologic Inc. manufacturer obtaining 59,311 mammograms, since they represented the majority of the data available. Images associated with malignant patients but with no annotation were discarded because they did not carry lesions information, thus reducing the usable data to 15,945 images. We automatically and manually discarded images with artifacts, clips, implants and corrupted images yielding a binary dataset composed as follows:

- Positive class: containing 5801 images with malignant findings selected from cases classified as malignant by either surgery, or biopsy, or previously classified as malignant. Each image may contain one or more of the following lesions: masses, calcifications, architectural distortions, focal asymmetries;
- Negative class: composed of 9895 images from women with normal breast.

This dataset has an imbalance of 1:1.7 whose effect on neural networks training can be considered negligible according to recent literature findings [17]. In Table 1 we report the detailed distribution of lesions for the positive class. Since an image is not uniquely associated with a lesion, we employ two methods for selecting images based on the type of findings: a MIXED approach in which an image is included when it contains at least an annotation of the specified lesion, and an EXCLUSIVE approach in which an image is included if it contains only annotations of the specified lesion.

### 2.2. Preprocessing

Before feeding the data to the networks, we performed a series of transformations as follows. First, the DICOM files were converted to 16-bit PNG format. Then, breast-air boundary was automatically segmented using fixed thresholding and images were cropped to remove as much background as possible. Segmentation and cropping were visually checked and manually corrected when needed. All the images were resized to the desired resolution, which was 1024×512 pixels in the first phase of the experimental study, see Section 2.4.1. Linear Normalization in the range [0,1] was applied to all the images and since we classify single-view mammograms, right view images were flipped obtaining the same orientation for the entire dataset. Finally we replicated the first channel three times simulating a three-channel color image in order to benefit from the use of pretrained networks that were originally trained on natural color images.

### 2.3. Network Architectures

In this section, we briefly discuss the convolutional and transformer architectures used in this work.

#### 2.3.1. ResNet

ResNet [26] is a family of VGG [40] inspired networks proposed in 2015 that won the ILSVRC-2015 competition. ResNet introduces a building block for residual learning that facilitates the training of deep models. It has been shown that adding layers to a CNN not only degrades the performance on the validation set but also on the training set. Instead of learning directly the mapping between input and output, the *residual block* learns only the residual function with respect to the identity mapping. This eases the learning task and at the same time alleviates the vanishing gradient problem, thus addressing the degradation in performance caused by adding layers and allowing deeper networks design.

#### 2.3.2. DenseNet

DenseNet [38] was introduced in 2017, the main idea of this design is to connect each layer with the others. The architecture is composed of *dense blocks* connected by means of *transition layers*. Inside a dense block the feature maps of a specific layer are used as input to all the following layers. In contrast to ResNet, concatenation instead of sum is used when joining the feature maps. Since concatenation is not possible with feature maps of different size, pooling is performed only in the transition block. DenseNet has several advantages: it alleviates the vanishing gradient problem, it strengthens feature propagation, it encourages feature reuse, and it substantially reduces the number of parameters.

#### 2.3.3. EfficientNet

Tan et al. [39] proposed a new method that uniformly scales resolution, depth and width of a convolutional network based on a so called *compound coefficient*. EfficientNet is a family of networks obtained by scaling a baseline CNN synthesized using a neural architecture search that optimizes both accuracy and FLOPS. EfficientNet achieved better accuracy and efficiency than previous CNNs on ImageNet classification.

#### 2.3.4. ConvNeXt

Inspired by Vision Transformer, Liu et al. [6] modify the architecture of a ResNet-50 towards the design of a hierarchical vision transformer (Swin) and adopted more recent training techniques, without introducing any attention-based module. The main architectural changes and design decisions are twofold. First, they applied a macro design consisting in changes of the number of layers in each block and in patchifying the input image. Second, they adopted grouped convolution, inverted bottleneck, large kernel size, and various layer-wise micro designs like GeLU instead of ReLU. They discover that a pure convolutional architecture can compete with state-of-the-art transformers suggesting that self-attention might not be the dominant factor that explains the competing performance and scalability of transformers.

#### 2.3.5. ViT

ViT [27] is the adaptation of the vanilla Transformer from NLP to computer vision. The Transformer works with an input that consists of a sequence of words (or *tokens*). The authors of ViT generate such sequence by splitting the input image into non-overlapping patches with a fixed size of 16×16 pixels. Each patch is linearly projected into a fixed sized space, and a *class token* is added to the sequence of embeddings. The sequence so obtained is provided to the transformer encoder which consists of alternating layers of multi-head self-attention and multi-layer perceptron (MLP) blocks. Layer normalization is applied before every block and residual connections after every block. The core of this and every other transformer architecture is the self-attention module that performs dot product attention between three different projections of the same input sequence. ViT achieved state-of-the-art performance on natural image classification on ImageNet but it was trained with a large private dataset consisting of >300 M images. This need of data can be explained by the lack of inductive biases, such as locality and spatial equivariance, that instead are present in convolutional networks.

#### 2.3.6. DeiT

Data efficient image Transformer (DeiT) [29] share the same architecture of ViT except for the addition of an extra token, called *distillation token*, to the sequences of embedding patches. This special token acts similarly to the class token and is given as input to a classifier called *distillation head*. During training, two loss function components are computed and then averaged: one between the label and the output of the classification head and the other between the output of a teacher network and the output of the distillation head. The authors also proposed an efficient training strategy for vision transformer in natural image domain based on the use of aggressive data augmentation and regularization techniques. In our work, we tested only the teacher-student training strategy relying on the distillation token, without adopting their data augmentation and regularization.

#### 2.3.7. Swin and SwinV2

Swin [30] is a hierarchical vision transformer that serves as general purpose backbone for computer vision. The characteristic of Swin is to organise images patches in windows of fixed size in which local self-attention is performed. Between two consecutive local attention steps, the windowing scheme is shifted of half the patch size to allow information flowing across windows. The overall architecture consists of 4 stages in which local attention and shifted local attention are performed a various number of times. After each stage, four adjacent patches are merged together using an embedding with double the patch size. Swin builds hierarchical feature maps by merging image patches in deeper layers and has linear computation complexity to input image size. This mechanism reintroduces some priors typical of CNNs.

SwinV2 has two main differences in the architecture compared to its predecessor. First, it uses an attention module with cosine function instead of the dot product and applies layer normalization after self-attention and MLP blocks. Second, positional embedding is achieved by a log-spaced continuous position bias method that relies on the use of a neural network with a fixed number of parameters that instead in Swin depended on the image size. These modifications allow SwinV2 to easily scale up capacity and resolution, and to surpass the performance of Swin on ImageNet. SwinV2 obtained state-of-the-art results in a variety of visual processing tasks: image classification with a top-1 accuracy of 84.0% on the ImageNet-V2 dataset, object detection with a 63.1/54.4 box/max AP on COCO test-dev, semantic segmentation with a 59.9 mIoU on ADE20K validation set, and video action classification where it achieved 86.8% top-1 accuracy on the Kinetics-400.

#### 2.3.8. NesT

NesT [32] is a hierarchical transformer proposed by Zhang et al. in 2022. NesT model stacks canonical transformer encoders to process non-overlapping image blocks individually. Each block is associated with a certain embedding sequence. Cross-block self-attention is achieved by nesting these transformers hierarchically and connecting them with an aggregation function. In particular, a convolution followed by layer normalization and max pooling was choosen as aggregation function.

### 2.4. Experimental Design

We randomly split the dataset into train and test sets according to a 80:20 ratio preserving the proportion between the classes. We maintain the same train-test split for all the experiments. In Table 2 we provide the detailed number of images in the test set for each lesion.

We employ transfer learning from ImageNet1K for all the architectures considered. The pretrained weights were obtained with an input resolution of 224×224 or 256×256 pixels. Specifically, we applied fine-tuning by loading the pretrained weights and allowing all layers to learn. When possible, we used the weights from Torchvision. For SwinV2 we utilized the official weights available at https://github.com/microsoft/Swin-Transformer (accessed on 29 November 2022) and for NesT the weights provided by the Timm library [41].

All the experiments were performed on a workstation running Ubuntu 18.04.3 LTS equipped with an Intel Xeon Silver 4110 CPU @ 2.10 GHz, 376 GB of RAM and one Nvidia A100 GPU with 80 GB of VRAM.

The experimental study was divided into two phases as follows. In the first phase, 33 distinct models were evaluated on the dataset built from OMI-DB while in the second phase, two models with similar performances, one convolutional and the other transformer-based, were compared as the input image resolution varied.

#### 2.4.1. Phase 1: Model Benchmarking

The selected models for comparison were the following: four versions of ResNet, namely ResNet-18, ResNet-34, ResNet-50 and ResNet-101, where the suffix represents the number of layers; four implementations of DenseNet, namely DenseNet-121, DenseNet-161, DenseNet-169 and DenseNet-201; eight EfficientNet models, from EfficientNet-B0 to EfficientNet-B7, where a higher number corresponds to a more complex model with a larger number of parameters; three versions of ConvNeXt, namely ConvNeXt-T, ConvNeXt-S, ConvNeXt-B where -T, -S and -B stay for tiny, small and base and refer to models with increasing complexity; three ViT models, namely ViT-T/16, ViT-S/16 and ViT-B/16 that refer to the tiny, small and base versions with a patch size of 16, small and tiny version are taken from DeiT with no distillation; two DeiT models, the tiny and small with distillation; six SwinV2 models, the tiny, small and base, each in two different configurations, with window sizes equal to 1/32 of the shortest side of the input resolution (SwinV2-*X*-W8) and 1/16 (SwinV2-*X*-W16); three NesT implementations, NesT-T, NesT-S, NesT-B. For training DeiT networks with knowledge distillation training strategy we used as teacher the EfficientNet-B3 model already fine-tuned on our dataset.

**Figure 2:**
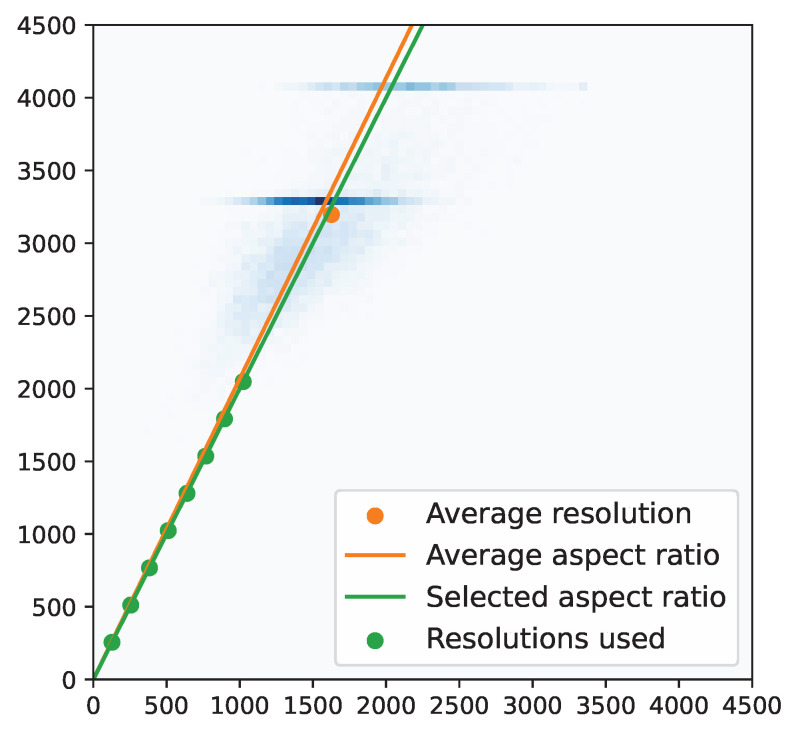
2D histogram of image resolutions. The orange line and circle represent the average of aspect ratios and the ratio between average height and width, respectively. The green circles indicate the resolutions used in this work.

The input resolution was fixed at 1024×512 pixels. This choice was motivated by a trade-off between performance and computational cost and because the 2:1 aspect ratio is similar to the average aspect ratio of the images after cropping, see Figure 2. The average of aspect ratios is 2.07:1 whereas the ratio between the average height and width is 1.97:1.

For NesT architectures, this resolution could not be used because they require a square input image. Thus, we chose the square resolution multiple of 32 which best approximated in number of pixels the input resolution 1024×512, obtaining 736×736 pixels. In this phase we ran each experiment twice and reported only the best performance. In this way, we mitigate the variability of the results due to the random initialization of the models and the random batching of stochastic gradient descent (SGD).

#### 2.4.2. Phase 2: Varying the Input Image Resolution

We selected two similar performing models from the best convolutional and transformer based family networks: EfficientNet-B0 and SwinV2-B-W8. We tested eight different input resolutions: 256×128, 512×256, 768×384, 1024×512, 1280×640, 1536×768, 1792×896 and 2048×1024. Note that like in Phase 1, the 2:1 aspect ratio is preserved for all the different input resolutions. Some lesions, such as masses, are visible starting from low resolutions, while small and sparse lesions such as calcifications require higher resolution to be detected. For these reasons, we have chosen a range of resolutions wide enough to observe the behavior of the two networks both in the case that a specific lesion is barely visible and in the case that it is well defined. Resolutions higher than 2048×1024 were very difficult to use due to limitations related mainly to memory but also to training time, and no performance improvements have been observed to suggest benefits from using higher resolutions.

#### 2.4.3. Hyper-Parameters

We employ SGD with a 0.9 momentum and a batch size equal to 4. Because of memory limitations, only for SwinV2-B-W8 we adopted a smaller batch size of 2 with an input resolution of 1792×896 and of 1 with an input resolution of 2048×1024. We chose a base learning rate of $10^{-3}$ and selected a Cosine annealing scheduler with warm restart at epoch 15 for a total of 30 epochs. The loss function used was the binary cross entropy.

### 2.5. Metrics

For each experiment, Accuracy, Sensitivity, Specificity, Matthews Correlation Coefficient (MCC) and Receiver Operating Characteristic (ROC) curves were computed for evaluating the performance. Accuracy is perhaps the most common performance measure for a classifier, consisting in the percentage of correctly classified samples over the total. MCC [42] is a correlation coefficient between prediction and true label, it produces a more informative and truthful score in evaluating binary classifications than accuracy giving a more realistic interpretation of classifier performance especially in the case of unbalanced datasets. The ROC curve shows the diagnostic ability of a binary classifier by plotting the Sensitivity over the False-Positive Rate (FPR) varying the threshold value. The area under the ROC curve (AUC) is a widely used metric that offers a simple way to summarize the overall performance of a model. In addition to these global metrics, we also computed per-lesion Sensitivity, AUC and MCC. These metrics were evaluated on the subset of test set containing images with a specific lesion, using both MIXED and EXCLUSIVE approaches for selecting the test subsets.

## 3. Results

### 3.1. Model Benchmarking

In Table 3 we provide the results obtained in the first phase of the experimental study. It can be observed that four network families, ResNet, DenseNet, EfficientNet and SwinV2 easily surpassed the others for all the metrics considered. We also note that ViT and DeiT performed similarly thus indicating that the knowledge distillation training strategy with EfficientNet teacher was not effective. Among the ConvNeXt models, only one (ConvNext-B) reached convergence during training, achieving a competing performance with respect to the other architectures considered. The best performing convolutional network was the EfficientNet-B3 with an Accuracy of 85.2% and an AUC of 90.2% whereas the best performing transformer network was the SwinV2-B-W8 with an Accuracy of 83.4% and an AUC of 88.7%. Among all transformers, SwinV2 family was the only one that could compete with common CNNs like ResNet, DenseNet and EfficientNet. In all experiments, the Specificity was always higher than the Sensitivity indicating that the networks were more likely to classify an image as negative, this is likely due to the class imbalance present in the dataset.

**Figure 3:**
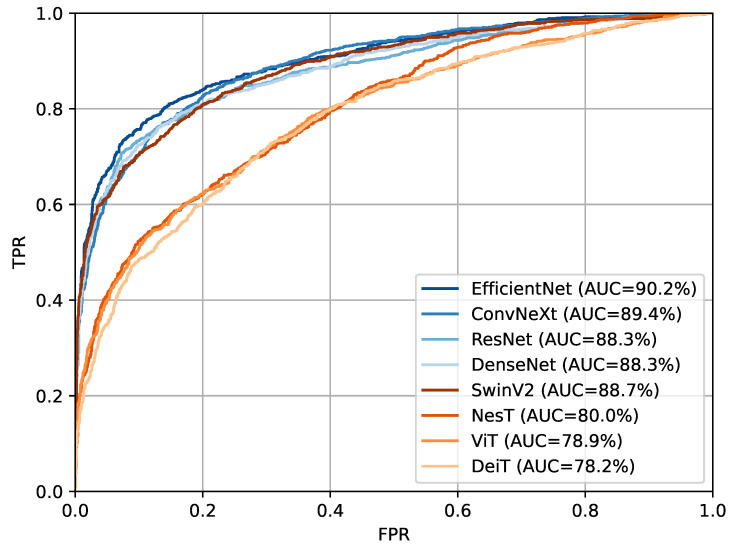
ROC curves of the best model for each of the eight families. In shades of orange are represented the convolutional models while in shades of blue the transformer-based models. In the legend the AUC value for each ROC curve is reported.

We report in Figure 3 the ROC curves of the best performing models for each family. This highlights a significant gap in performance between two groups, the convolutional models together with the SwinV2, and the other transformer models NesT, ViT, and DeiT.

**Figure 4:**
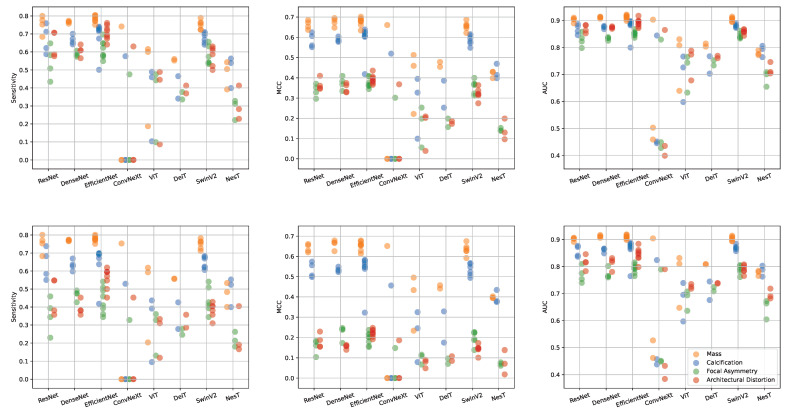
Performance per-lesion for each combination of metrics (Sensitivity, MCC, AUC) and image selection approach (MIXED **top** row, EXCLUSIVE **bottom** row). On the x-axis results are grouped by network families whereas on the y-axis the metric considered is reported. Each color represents a specific lesion (yellow:mass, blue:calcification, green:focal asymmetry, red:architectural distortion).

In Figure 4 we provide a per-lesion analysis of the performances of the 33 models for each of the 6 combinations of metrics (Sensitivity, MCC and AUC) and image selection methods (MIXED and EXCLUSIVE). The best results are obtained on images with masses, then on calcifications, and finally on focal asymmetries and architectural distortion with similar performances. This order reflects the number of images for each lesion in the dataset suggesting that the difference in performance is due to dataset composition and not to the particular characteristics of the lesions. There seems to be no relationship between lesion sparsity and the two visual processing paradigms, convolution and attentional. NesT is the only architecture that classifies calcifications better than masses. Also note how the metrics calculated using the MIXED approach are generally lower to those calculated using the EXCLUSIVE approach. This is particularly evident for architectural distortions and focal asymmetries while the difference is almost zero in the case of masses.

### 3.2. Varying the Input Image Resolution

**Figure 5:**
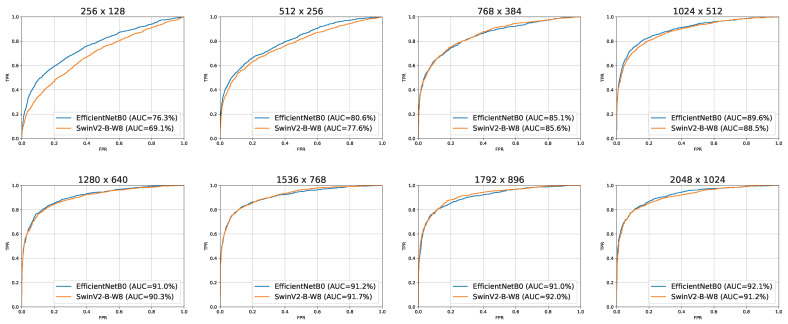
ROC curves of EfficientNet-B0 and SwinV2-B-W8 for all input resolutions used. In the legend the AUC value for each ROC curve is reported.

In Table 4 are shown the results obtained by varying the input image resolution from 256×128 to 2048×1024 with EfficientNet-B0 and SwinV2-B-W8 as described in Section 2.4.2. There is a significant increase in performance as the resolution increases up to 1280×640, whereas after 1536×768 the performances do not vary significantly. At lower resolutions, 256×128 and 512×256, there is a clear advantage of EfficientNet-B0 whereas as the resolution increases the two models perform similarly. This is further highlighted by the ROC curves reported in Figure 5. The best AUC for SwinV2-B-W8 model was 92.0% at 1792×896, this value is comparable to the best AUC obtained from EfficientNet-B0 of 92.1% at 2048×1024.

**Figure 6:**
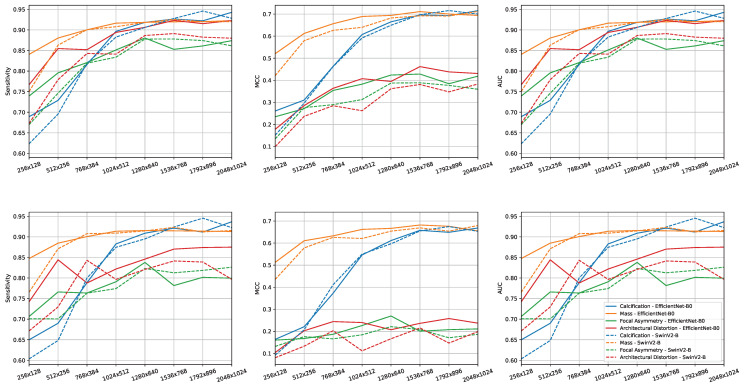
Performance per-lesion in experiments as the resolution changes for each combination of metrics (Sensiticity, MCC, AUC) and image selection approach (MIXED **top** row, EXCLUSIVE **bottom** row). On x-axis the input resolution and on y-axis the value for the metric considered. Continues and dashed line indicate respectively EfficientNet-B0 and Swin-B-W8. Colors represent different lesions (yellow-mass; blue-calcification; green-focal asymmetry; red-architectural distortion).

In Figure 6 we report the per-lesion performance plots by varying the input image resolution for each of the 6 combinations of metrics (Sensitivity, MCC and AUC) and image selection methods (MIXED and EXCLUSIVE). It can be observed how the classification of images with calcifications is affected more than other lesions by the resolution of the input image. At 256×128 resolution the difference in MCC between images containing masses and images containing calcifications, averaged by network and image selection method, was 0.305 while at 1536×768 was 0.012. A similar behavior can be observed for the other metrics. Swin-B-W8 and EfficientNet-B0 behave similarly with all lesions at higher resolutions, with a notable difference at low resolutions.

## 4. Discussion

### 4.1. Transformers or CNNs?

From the results of the first phase of experiments we can observe that CNNs trained more easily and with better results compared with transformer networks. Specifically, the more recent convolutional networks were faster, more efficient, and more accurate than older ones. Nevertheless, the most recent CNN tested, ConvNeXt, did not always converge. This can be imputed to the process of “modernization” towards the architecture of Swin Transformer that brings also the difficulty in training typical of vision transformers and the high sensitivity to model initialization and hyper-parameters. SwinV2 competed with CNNs at the cost of longer training times and higher memory usage. SwinV2-B-W8 required 22 GB of memory, while EfficientNet-B0 only 6 GB. The superior performance of SwinV2 compared to the other transformer networks can be due to the reintroduction of the locality bias, although to a lesser extent than convolutional networks.

In the context of our study, one possible reason for the lack of competitiveness of transformer-based networks with respect to CNNs could be the small size of the datasets used for training when compared to those used in other applications, such as for natural image classification. ViT was originally trained on a dataset with 300 M of images and SwinV2 on ImageNet1K consisting of 1.2 M images. In the medical field, including mammography analysis, it is difficult to retrieve a comparable amount of images.

### 4.2. Lesion-Based Analysis

No different behavior was observed in classifying images with different types of lesions by the two visual processing paradigms. This indicates that locality of CNNs is not a limitation, also for the classification of images with sparse lesions like calcifications. Further, it suggests that it is not necessary to capture long-range dependencies in the first layers. To support this, we recall that most of the lesions are well localized including calcifications that are often grouped in a region of the breast. Thus, the locality bias of CNNs which was reintroduced in Swin-V2 might play a crucial role.

As was to be expected, it was easier to classify images with more than one lesion as there are different objects and elements that the networks can use to make the prediction. Among all the architectures, NesT was the only one that obtained better results on calcifications rather than masses, this could be due by either the different input resolution used or the particular type of hierarchical attention and merging strategy adopted.

### 4.3. Resolution-Based Analysis

As the input resolution increased, the SwinV2 model closed the performance gap with respect to the convolutional counterparts. We believe this might be due to two related factors. First, the local receptive field enforces the CNN to attain a global view of the input only in the very last layers. On the other hand, SwinV2 windows size scales with input resolution. Vanilla Vision Transformer is practically unusable at these high resolutions because of the computational complexity of global attention. For resolutions greater than 2048×1024, it is difficult to expect an increase in performance for both EfficientNet and SwinV2 since the results from 1536×768 to 2048×1024 are very close to each other. Other medical fields that make use of very high-resolution imaging could benefit from the Swin transformers scaling capability. In the field of mammography image analysis, many works adopt an input resolution of 224×224, we recommend using a resolution of the input images after cropping greater than 1200×600. This also brings benefits in the classification of large lesions such as masses.

### 4.4. Explainability

In the context of medical image analysis, including mammogram classification, Explainable Artificial Intelligence (XAI) is of outmost importance. *Class Activation Map* (CAM) [43] methods and their variant *Grad-CAM* [44] and *Grad-CAM++* [45] were specifically designed for CNNs and are the most widely adopted XAI methods in medical imaging. They are based on a per-class weighted linear sum of visual patterns present at various spatial locations in an image and produce heatmaps representations that indicate which regions of the input image were most important for CNN’s decisions. Recently, there are initial attempts to use Grad-CAM on transformer architectures but their effectiveness is still on debate [46,47]. However, thanks to the attention mechanism, transformers are intrinsically able to support explanations based on the inspection of the weights in the attention matrices, like the *Attention Rollout* [48]. In addition, hierarchical transformers can benefit from ad hoc XAI methods, for example NesT’s *GradCAT* relies on the particular tree hierarchy of the architecture by finding the path from a leaf node to the root node that contributes most to the output of the network.

### 4.5. Limitations

This work carries with it some limitations: (i) we reported for each method the best performance from two runs, however this is not sufficient for a statistical analysis of the results; (ii) we performed binary classification, but multi-label training, using the four types of lesions as classes, might give additional indications on how transformers and CNNs process mammography images; (iii) we did not perform hyper-parameter optimizations, this could potentially unveil additional performance improvements in a more practical scenario; (iv) customizing the architectures in the case of mammogram analysis could bring out the true potential of the two visual processing paradigms, whereas in this work we used each model “as is”; and (v) we did not experimentally analyze the explainability of our transformer and convolutional models.

## 5. Conclusions

Vision Transformers are emerging as powerful architectures capable of learning long-range dependencies. In this work we compared different models from both convolutional and attentional paradigms with the aim of verifying the effectiveness of transformer-based architectures for mammography image classification. We conclude that transformer-based architectures did not perform as well as in natural image application when compared to CNNs. The hierarchical SwinV2 transformer was the only architecture that competed with CNNs indicating an advantage in using networks that incorporate a locality bias. We recommend, especially in case of limited time and memory resources, to use modern convolutional networks instead of vision trasformers. The promising results of SwinV2 transformer should be further investigated, for example by using larger datasets and customizing its architecture that is optimized for natural image analysis.

## Figures and Tables

**Table 1:** Numbers of images for each lesion using both MIXED and EXCLUSIVE method for image selection.

Lesion Type	Number of Images with MIXED Method	Number of Images with EXCLUSIVE Method
Mass	3175	2471
Calcification	2563	1833
Focal Asymmetry	593	290
Architectural Distortion	499	238
Total	5801	

**Table 2:** Numbers of images for each lesion using MIXED and EXCLUSIVE method in the test set. In parentheses the percentage with respect to the entire dataset.

Lesion Type	Number of Images with MIXED Method in Test Set	Number of Images with EXCLUSIVE Method in Test Set
Mass	630 (19.84%)	482 (19.51%)
Calcification	531 (20.72%)	379 (20.62%)
Focal Asymmetry	122 (20.57%)	61 (21.03%)
Architectural Distortion	92 (18.44%)	42 (17.65%)
Total	1160	

**Table 3:** Results of the experiments for each of the 33 architectures used. In bold the highest value of Accuracy, MCC and AUC. Column TpE indicates the training time per epoch in minutes.

Model	TpE	Accuracy	MCC	AUC	Sensitivity	Specificity
ResNet18	4	80.9%	58.2%	85.8%	60.3%	93.0%
ResNet34	5	82.3%	61.3%	87.5%	65.7%	92.1%
ResNet50	8	83.7%	64.6%	88.8%	75.4%	88.5%
ResNet101	13	84.3%	65.7%	88.3%	71.6%	91.7%
DenseNet-121	8	82.7%	62.2%	88.3%	70.9%	89.5%
DenseNet-161	14	83.1%	63.1%	88.7%	68.3%	91.9%
DenseNet-169	10	83.7%	64.4%	88.3%	69.1%	92.3%
DenseNet-201	13	83.6%	64.1%	88.0%	68.0%	92.7%
EfficientNet-B0	4	84.2%	65.6%	89.5%	71.9%	91.5%
EfficientNet-B1	5	83.9%	64.9%	89.0%	69.8%	92.2%
EfficientNet-B2	5	84.2%	65.6%	89.2%	72.3%	91.2%
**EfficientNet-B3**	7	**85.2%**	**67.7%**	**90.2%**	74.5%	91.5%
EfficientNet-B4	9	79.5%	55.0%	84.8%	62.1%	89.8%
EfficientNet-B5	11	84.2%	65.5%	88.5%	71.7%	91.5%
EfficientNet-B6	12	84.5%	66.2%	89.3%	74.9%	90.0%
EfficientNet-B7	16	83.7%	64.5%	89.1%	74.2%	89.2%
ConvNeXt-T	16	63.0%	0.0%	46.9%	0.0%	100.0%
ConvNeXt-S	19	63.0%	0.0%	47.2%	0.0%	100.0%
ConvNeXt-B	22	83.1%	63.4%	89.4%	75.1%	87.7%
ViT-T/16	8	73.2%	40.1%	76.2%	50.7%	86.4%
ViT-S/16	17	75.7%	45.9%	78.9%	53.0%	89.0%
ViT-B/16	31	65.5%	17.3%	62.7%	14.6%	95.4%
DeiT-Ti	10	71.8%	36.4%	75.1%	43.1%	88.7%
DeiT-S	19	74.5%	43.0%	78.2%	48.5%	89.7%
SwinV2-T-W8	16	81.9%	60.3%	87.7%	67.4%	90.3%
SwinV2-T-W16	23	82.1%	60.9%	88.3%	68.5%	90.1%
SwinV2-S-W8	26	82.5%	61.8%	88.1%	70.8%	89.3%
SwinV2-S-W16	73	83.0%	63.0%	88.1%	72.4%	89.2%
SwinV2-B-W8	28	83.4%	63.8%	88.7%	65.2%	94.1%
SwinV2-B-W16	92	83.4%	63.7%	88.4%	69.3%	91.7%
NesT-T	33	75.6%	45.6%	78.5%	50.0%	90.6%
NesT-S	54	76.0%	46.7%	80.0%	51.1%	90.7%
NesT-B	77	73.5%	40.9%	73.3%	38.5%	94.0%

**Table 4:** Results of the experiments varying the input resolution. In bold the highest value of Accuracy, MCC and AUC for both networks tested. Column TpE indicates the training time per epoch in minutes.

Models	Input Resolution	TpE	Accuracy	MCC	AUC	Sensitivity	Specificity
EfficientNet-B0	256×128	3	73.7%	41.4%	76.3%	51.4%	86.9%
	512×256	3	77.0%	49.0%	80.6%	55.1%	89.8%
	768×384	4	80.3%	56.6%	85.1%	60.3%	92.0%
	1024×512	5	84.3%	65.8%	89.6%	72.8%	91.0%
	1280×640	6	85.7%	68.9%	91.0%	76.6%	91.0%
	1536×768	7	**86.3%**	**70.2%**	91.2%	75.2%	92.8%
	1792×896	9	86.1%	69.8%	91.0%	75.9%	92.1%
	2048×1024	12	86.0%	69.5%	**92.1%**	74.3%	92.9%
SwinV2-B-W8	256×128	9	69.9%	31.2%	69.1%	34.7%	90.6%
	512×256	10	76.0%	46.6%	77.6%	53.5%	89.1%
	768×384	20	80.2%	56.7%	85.6%	65.3%	89.0%
	1024×512	28	82.7%	62.2%	88.5%	71.6%	89.1%
	1280×640	64	84.7%	66.7%	90.3%	73.5%	91.3%
	1536×768	84	86.0%	69.5%	91.7%	76.5%	91.6%
	1792×896	139	86.0%	69.5%	**92.0%**	76.5%	91.5%
	2048×1024	205	**86.1%**	**69.9%**	91.2%	76.2%	92.0%

## Data Availability

The OMI-DB dataset [49] employed in the current study is publicly available at https://medphys.royalsurrey.nhs.uk/omidb/ (accessed on 29 November 2022). The list of images that we extracted and used in our experiments are available from the corresponding author upon reasonable request.
